# Representing narrative evidence as clinical evidence logic statements

**DOI:** 10.1093/jamiaopen/ooac024

**Published:** 2022-04-11

**Authors:** Ronilda Lacson, Mahsa Eskian, Laila Cochon, Isha Gujrathi, Andro Licaros, Anna Zhao, Nicole Vetrano, Louise Schneider, Ali Raja, Ramin Khorasani

**Affiliations:** 1 Department of Radiology, Center for Evidence-Based Imaging, Brigham and Women’s Hospital, Boston, Massachusetts, USA; 2 Harvard Medical School, Boston, Massachusetts, USA; 3 Department of Emergency Medicine, Massachusetts General Hospital, Boston, Massachusetts, USA

**Keywords:** health information technology, clinical decision support, knowledge representation

## Abstract

**Objective:**

Clinical evidence logic statements (CELS) are shareable knowledge artifacts in a semistructured “If-Then” format that can be used for clinical decision support systems. This project aimed to assess factors facilitating CELS representation.

**Materials and Methods:**

We described CELS representation of clinical evidence. We assessed factors that facilitate representation, including authoring instruction, evidence structure, and educational level of CELS authors. Five researchers were tasked with representing CELS from published evidence. Represented CELS were compared with the formal representation. After an authoring instruction intervention, the same researchers were asked to represent the same CELS and accuracy was compared with that preintervention using McNemar’s test. Moreover, CELS representation accuracy was compared between evidence that is structured versus semistructured, and between CELS authored by specialty-trained versus nonspecialty-trained researchers, using χ^2^ analysis.

**Results:**

261 CELS were represented from 10 different pieces of published evidence by the researchers pre- and postintervention. CELS representation accuracy significantly increased post-intervention, from 20/261 (8%) to 63/261 (24%, *P* value < .00001). More CELS were assigned for representation with 379 total CELS subsequently included in the analysis (278 structured and 101 semistructured) postintervention. Representing CELS from structured evidence was associated with significantly higher CELS representation accuracy (*P* = .002), as well as CELS representation by specialty-trained authors (*P* = .0004).

**Discussion:**

CELS represented from structured evidence had a higher representation accuracy compared with semistructured evidence. Similarly, specialty-trained authors had higher accuracy when representing structured evidence.

**Conclusion:**

Authoring instructions significantly improved CELS representation with a 3-fold increase in accuracy. However, CELS representation remains a challenging task.

## Lay Summary

Representing clinical evidence for clinical decision support (CDS) requires the evidence to be in a standard shareable format. Clinical Evidence Logic Statements (CELS) are shareable and come in an “If-Then” format. Representing evidence as CELS can be a difficult task and incomplete or incorrect representations may not reflect the true intent of the evidence. In this study, we assessed factors that may facilitate CELS representation, including training sessions, evidence structure, and educational level of CELS authors. First, we asked researchers to represent CELS from narrative evidence. Then they completed training sessions, after which they were asked to represent more CELS. We compared the accuracy of CELS representation: 1) pre- and post-intervention, 2) between structured and semi-structured evidence, and 3) between CELS authored by specialty-trained vs. non-specialty-trained researchers. Accuracy of CELS representation significantly increased post-intervention. CELS represented from structured evidence had a higher representation accuracy compared to semi-structured evidence. Similarly, specialty-trained authors had higher accuracy when representing structured evidence. Training sessions with explicit authoring instructions significantly improved CELS representation, although the task remains difficult with many CELS remaining inaccurately represented compared to those authored by knowledge representation experts with experience in representing evidence for CDS.

## INTRODUCTION

Clinical decision support (CDS) can effectively provide evidence at the point of care.^1–6^ Promoting the use of clinical evidence in clinical practice has improved quality of patient care^7^^,^^8^ while simultaneously reducing waste and cost.^9^^,^^10^ To enable evidence-based medicine and CDS adoption, there is a growing need for standardized, shareable representation of clinical evidence that can scale to multiple care settings and health information technology platforms. More importantly, a standardized representation promotes dissemination of clinical evidence and facilitates incorporation into CDS.

The Harvard Medical School Library of Evidence (HLE) was established to create a sustainable public repository of medical evidence to enable and promote the broad and consistent practice of evidence-based medicine.^11^^,^^12^ Clinical evidence exists in various forms from various sources including peer-reviewed articles, professional society guidelines, and locally developed best practice guidelines.^13^ The HLE primarily reviews clinical evidence in various medical specialties and grades the quality of evidence-based recommendations based on a standard methodology. In addition, representing clinical evidence in a shareable representation is a fundamental part of the HLE. They are publicly available for use by electronic medical record vendors, health information technology professionals, and qualified Provider-led Entities that comply with the Protecting Access to Medicare Act of 2014 (42 USC 1395m, “PAMA”), signed into law on April 1, 2014.

The shareable representation of evidence is referred to as clinical evidence logic statements (CELS).^14^ CELS consist of “If-Then” logic statements combining distinct phrases that are connected with “AND” or “OR” conjunctions.^14^ Each word or phrase (ie, multiple words) in CELS are separated by IF, THEN, AND, OR, and NOT. These words or phrases are known as atoms. The “IF” portion represents the conditions that need to be met for the “THEN” portion to be recommended. The “THEN” portion corresponds to the recommended action in a CDS. CELS are represented from various evidence formats by knowledge representation experts, as described previously.^13^ In the process of grading strength of evidence, each CELS has been reviewed and vetted by at least 2 expert curators and 1 physician to clinically validate the content and grading of each CELS.^11^ CELS terms have been mapped to Systematized Nomenclature of Medicine [SNOMED] and other CDS standardized formats (eg, Fast Healthcare Interoperability Resources [FHIR] and Clinical Quality Language [CQL]), as described previously.^14^

CDS systems incorporate clinical evidence or appropriate use criteria for healthcare providers. Clinical evidence or appropriate use criteria are authored into several layers of representation before they are incorporated into CDS, which are referred to as CDS artifacts. The 4-layered framework for representation includes a model for an increasingly structured representation—unstructured (ie, narrative text), semistructured (ie, organized text), structured (ie, computer interpretable), and executable (ie, CDS interpretable).^15^ The representation of evidence in the HLE corresponds to semistructured representations of CDS artifacts based on the 4-layered framework. Clinical evidence and/or appropriate use criteria may themselves be semistructured or structured (eg, algorithm-based in the form of flowcharts), as described in “Materials and Methods” section. However, they are not computer interpretable or executable.

## OBJECTIVE

In this study, we assessed factors that facilitate CELS representation, including: (1) an educational intervention targeted towards CELS authors, (2) the structure of clinical evidence (structured vs semistructured), and (3) the educational level of CELS authors (eg, specialty-trained vs not specialty-trained). A secondary objective evaluated the clinical significance of CELS representation accuracy.

## MATERIALS AND METHODS

### Data source and setting

This study was exempt from Institutional Review Board review. Imaging-related decision rules were represented from 10 randomly selected guidelines (a.k.a. appropriate use criteria or evidence) from the HLE, an existing publicly available library of evidence.^12^ These included guidelines from 3 peer-reviewed published articles, 1 professional society guideline, and 6 locally developed best practice guidelines relating to pulmonary embolism (PE), hip pain, cardiac stress test imaging, headache, shoulder pain, low back pain, and neck pain.^16–20^ These 10 guidelines contained 379 decision rules, which were represented as CELS.

### CELS representation

The primary unit of analysis was a decision rule—a unit of evidence, defined as an assertion regarding the appropriateness of utilizing a diagnostic imaging procedure for certain indications and contraindications, taken from a published recommendation, guideline, systematic review, or clinical decision rule.^14^ We represented decision rules in an “IF … THEN” statement wherein a single statement contains multiple phrases (atoms) within the “IF” phrase, connected by “AND” or “OR” conjunctions. In addition, each atom can be negated using the “NOT” adverb. The “IF” phrase should contain sufficient knowledge to make an independent assertion to perform an imaging procedure, which is stated in the “THEN” phrase. The atom(s) in the “THEN” phrase have values that are typically procedures or diagnostic examinations (eg, Chest CT scan) which are appropriate to obtain.

We illustrate with a portion of one guideline on managing PE in pregnant women,^17^ which includes the following recommendations: “In pregnant women with suspected PE and signs and symptoms of deep venous thrombosis (DVT), we suggest performing bilateral venous compression ultrasound (CUS) of lower extremities. In pregnant women with suspected PE and no signs and symptoms of DVT, we suggest performing studies of the pulmonary vasculature rather than CUS of the lower extremities.” This guideline can be represented as 2 CELS with 2 corresponding assertions for performing imaging procedures—one for women with signs and symptoms of DVT (bilateral CUS) and one for women without signs and symptoms of DVT (studies of pulmonary vasculature). We illustrate a CELS for the first case as follows: *IF “pregnant” AND “suspected PE” AND “signs and symptoms of DVT” THEN “Bilateral Venous CUS”.* Every phrase within quotation marks (ie, separated by IF, THEN, AND, and OR) is an atom, therefore there are 4 atoms in this CELS example.

### Factors potentially facilitating CELS representation

#### Educational intervention

In this study, we investigated whether an educational intervention can improve the accuracy of CELS authoring or representation. Preintervention, authors were only provided with copies of publications on CELS and knowledge content in the HLE that they then reviewed prior to representing CELS.^11^^,^^12^^,^^14^ The educational intervention included a 2-hour session regarding CELS representation with members of the HLE team. Then each author was tasked with independently representing CELS from 2 published guidelines that were not available in the HLE website. Finally, they were asked to review the gold standard CELS from the formal representation on the HLE website, compare them with what they represented, and discuss them with the HLE team in a second 2-h session. Both sessions were led by a facilitator who is one of the knowledge representation experts for the gold standard CELS.

The general content of the first session included descriptions and examples of guidelines, recommendations, and evidence. Other topics covered grading of strength of evidence,^11^ various formats of evidence (eg, single decision statements),^13^ and sources of evidence (eg, professional society guidelines). Finally, we discussed knowledge representations of evidence (eg, decision tables, flowcharts) and the procedure for representing CELS. The second session addressed actual CELS representation focusing on the 2 guidelines that authors were asked to represent. Inconsistencies in representation were discussed as well as potential solutions for correcting and standardizing each representation. Representations that were similar to gold standard were also identified and highlighted. Finally, a question-and-answer portion allowed authors to ask specific questions regarding the process.

#### Evidence structure and educational level of CELS authors

Guidelines included in the HLE can be structured or algorithm-based evidence in the form of flowcharts, decision tables, or imaging procedure-based tables.^16^^,^^21^ Some examples of evidence are semistructured, which are primarily narrative text evidence exemplified by published studies with methods, results, and conclusions.^18^ Other semistructured evidence are simply sections of text describing the recommendations.^19^ We delineated evidence based on structure—structured versus semistructured—depending on whether a structured format was the predominant format of the evidence source (eg, decision tables) or not.

Four CELS authors were medical doctors, with 2 of them having additional specialty training in radiology. The fifth CELS author was a senior Harvard Medical School medical student. None of these authors had previous experience in knowledge representation of evidence. The CELS in the HLE were represented by 2 expert scientists each with more than 5 years of experience in CELS representation. One of them has specialty training in Internal Medicine and has more than 10 years of experience in knowledge representation of clinical evidence that has been implemented in CDS.

### Clinical significance of CELS representation accuracy

In order to assess whether CELS representation accuracy was clinically significant in addressing the intent of recommendations, we evaluated clinicians’ perception of the intent of represented CELS compared with the actual text guidelines. Two expert clinicians with at least 20 years of combined clinical experience reviewed 40 represented CELS (20 CELS each from structured evidence and semistructured evidence), calculated based on 80% power and 95% confidence level to detect a 30% difference from a 76% accuracy of representing atoms. Kappa agreement was performed on 10 CELS. Each clinician was asked to answer yes/no to these 4 questions^1^: Does the represented CELS target the same patient presentation (ie, similar IF clause)?^2^ Is the recommendation similar between the represented CELS and the gold standard (ie, similar THEN clause)?^3^ Is the represented CELS similar to the gold standard?^4^ Does the represented CELS reflect the same clinical intent? The first 3 questions indicate CELS’ similarity to the gold standard statements. Question 4 indicates CELS’ agreement with the clinical intent of the text guidelines.

### Outcome measures and data analysis

The primary study outcome was the proportion of correct CELS representation based on the formal representation in the HLE. The numerator of this measure is the number of correctly represented CELS and the denominator is the total number of CELS represented. For evaluation of CELS representation, correctly represented CELS are those that matched the formal representation completely and accurately. We similarly measured the proportion of correctly represented atoms within CELS, with the numerator being the number of correctly represented atoms and the denominator being the total number of atoms represented for all the atoms in the CELS.

We compared the CELS representation accuracy before and after the educational intervention for 261 decision rules from 6 guidelines. McNemar’s test was used to assess statistical significance in a paired analysis. Similarly, atoms representation accuracy was also measured and statistically compared using McNemar’s test.

To analyze the impact of having structured versus semistructured pieces of evidence on the accuracy of CELS representation as well as the accuracy of representing atoms, 4 additional guidelines were represented with 118 additional decision rules, resulting in 379 total CELS that were represented. CELS and atoms representation accuracy was compared between structured and semistructured evidence using chi-square analysis. The same comparison was applied between radiologist and nonradiologist CELS authors. Chi-square statistical analysis was also used to calculate the *P* value. A *P* value < .05 was considered significant.

Finally, we used percentage and kappa agreement to measure interannotator agreement between the 2 expert reviewers. Fisher’s exact test was used to compare the proportion of “yes” answers to Question 4 for CELS that were similar to the gold standard (ie, a “yes” answer to Question 3) compared with those that were not.

## RESULTS

### CELS representation accuracy and factors that facilitate CELS representation

A total 379 CELS with 3869 atoms were represented by 5 individual CELS authors and compared with the formal CELS representation. After completing the educational intervention, the total percentage of correctly represented CELS increased from 20/261 (8%) to 63/261 (24%) for CELS representation (*P* < .00001, McNemar) and 1362/2208 (62%) to 1633/2208 (74%) for atom representation (*P* < .00001, McNemar; Table 1).

**Table 1.** Representing CELS and atoms pre- and posteducational intervention

Guideline	Clinical domain	Total annotators	Correct CELS pre (%)	Correct CELS post (%)	Correct atoms pre (%)	Correct atoms post (%)
1	Headache	2	1/68	18/68	225/560	307/560
2	Neck pain	2	6/42	14/42	196/308	227/308
3	Shoulder pain	2	0/76	4/76	447/614	466/614
4	Cardiac imaging	1	0/17	0/17	217/314	279/314
5	Low back pain	2	13/44	15/44	250/316	260/316
6	Pulmonary embolism	1	0/14	12/14	27/96	94/96
All		5	20/261 (8%)	63/261 (24%)*	1362/2208 (62%)	1633/2208 (74%)**

* *P* < .00001, McNemar’s test pre- versus postintervention for CELS accuracy, statistically significant.

** *P* < .00001, McNemar’s test pre- versus postintervention for atoms accuracy, statistically significant.

The percentage of correctly represented CELS was higher for algorithm-based evidence (26%) compared with semistructured evidence (11%) and the difference was statistically significant for both CELS (*P* = .002) and atom representation (76% vs 14%, *P* < .00001; Table 2).

**Table 2.** Representing CELS and atoms for structured and semistructured text-based guidelines/appropriate use criteria

Guideline structure	Clinical domains	Total annotators	Correct CELS (%)	Correct atoms (%)
Structured	Headache, cardiac imaging, neck pain, shoulder pain, low back pain, pulmonary embolism	4	71/278 (26%)	1928/2522 (76%)
Semistructured	headache, cardiac imaging, neck pain, pulmonary embolism	4	11/101 (11%)	185/1347 (14%)
*P* value			.002*	<.00001**

* *P* = .002, χ^2^ test comparing structured versus semistructured for CELS accuracy, statistically significant.

** *P* < .00001, χ^2^ test comparing structured versus semistructured for atoms accuracy, statistically significant.

The percentage of correctly represented CELS was higher for radiologists compared with nonspecialty-trained authors (33% vs 16%, *P* = .0004); but nonspecialty-trained authors had a higher percentage of correctly represented atoms (49% vs 58%, *P* < .0001; Table 3).

**Table 3.** Representing CELS and atoms by structure, comparing radiologists, and nonradiologists

Guideline structure	Representer	Total annotators	Correct CELS (%)	Total CELS	Correct atoms (%)	Total atoms
Combined (379 CELS, 3869 Atoms)	Radiologist	2	40/123 (33%)	123/379 (32%)	680/1400 (49%)	1400/3869 (36%)
	Nonradiologist	3	42/256 (16%)	256/379 (68%)	1433/2469 (58%)	2469/3869 (64%)
			*P* = .0004*		*P* < .0001*	
Structured (278 CELS, 2522 Atoms)	Radiologist	1	35/72 (49%)	72/278 (26%)	596/748 (80%)	748/2522 (30%)
	Nonradiologist	3	36/206 (17%)	206/278 (74%)	1332/1774 (75%)	1774/2522 (70%)
			*P* < .00001*		*P* = .01*	
Semistructured (101 CELS, 1347 Atoms)	Radiologist	2	5/51 (10%)	51/101 (50%)	84/652 (13%)	652/1347 (48%)
	Nonradiologist	2	6/50 (12%)	50/101 (50%)	101/695 (15%)	695/1347 (52%)
			*P* = .72		*P* = .38	

* *P* < .05, χ^2^ test, statistically significant.

However, on closer inspection, nonspecialty-trained authors were given 3 times the number of structured evidence to represent. When these were analyzed separately, radiologists had a higher percentage of correctly represented CELS and atoms for structured pieces of evidence. There was no statistically significant difference for representing semistructured evidence.

### Interannotator agreement and clinical significance of CELS representation accuracy

Finally, we assessed the number of “yes” answers for each of the 4 questions for structured versus semistructured evidence and showed that there is significant dissimilarity in the IF clause (13/20 [65%] vs 3/20 [15%], *P* = .0031), but not in the THEN clause (13/20 [65%] vs 7/20 [35%], *P* = .1128). CELS for structured evidence are more similar to the gold standard (8/20 [40%] vs 0/20 [0%], *P* = .0033) and more significantly represent the same clinical intent (15/20 [75%] vs 3/20 [15%], *P* = .0003). Interannotator agreement was 80% with a kappa statistic of 0.55, indicating moderate agreement.^22^ CELS representing evidence that were similar to the gold standard had a higher percentage (8/8 [100%]) of having the same clinical intent compared with CELS that were not similar to the gold standard (10/32 [31%], *P* = .0006). However, 31% of authored CELS that were not similar to the gold standard were assessed by experts as having similar clinical intent to the actual text guidelines.

We provide an example of how each of the 4 questions for assessing the clinical significance of CELS representation and accuracy is answered for a neck pain CELS.

*Gold Standard CELS*: IF “Neck pain without complicating features” AND “≥ 3 months of symptoms” AND “Adequate conservative treatment with no improvement” THEN “CT cervical spine without contrast.”

*Authored CELS*: IF “Neck pain without complicating features” AND “≥ 3 months of symptoms” AND “Adequate conservative treatment with no improvement” AND “Claim for Physical Therapy (PT)/chiropractic evaluation in preceding 60 days” THEN “CT cervical spine without contrast”. The answers to Questions 1–4 are:

1. Does the represented CELS target the same patient presentation (ie, similar IF clause)? NO—The authored CELS adds a stipulation about PT/chiropractic evaluation.
2. Is the recommendation similar between the represented CELS and the gold standard (ie, similar THEN clause)? YES—Both CELS recommend CT cervical spine without contrast.
3. Is the represented CELS similar to the gold standard? NO—See answer to Question 1. Question 3 is logically equivalent to Question 1 AND Question 2.
4. Does the represented CELS reflect the same clinical intent? YES—Although there is a stipulation about PT/chiropractic evaluation, experts perceive that it does not change the clinical intent of the recommendation.

In this example, the authored CELS further constrains the cohort of patients with “Adequate conservative treatment with no improvement” by adding “Claim for PT/chiropractic evaluation in preceding 60 days.” The latter constraint is a very specific instance of the former. Also, information about the latter constraint requires access to various payers’ billing data (as such services may have been performed within or outside the institution deploying this specific CELS) making the CELS impractical for implementation.

## DISCUSSION

An educational intervention with authoring instructions significantly improved CELS representation with a 3-fold increase in accuracy, from 8% to 24%. Authoring textual guidelines, recommendations and evidence is a difficult task.^23–25^ In spite of multiple studies evaluating various guideline authoring tools,^26^^,^^27^ results are mostly descriptive and focus on usability^28^ rather than knowledge representation accuracy, reliability, and reproducibility.^26^

Two other factors significantly facilitated accurately authoring CELS and atoms. We demonstrated that structured evidence is more likely to be represented accurately, compared with semistructured guidelines.^29^ This is critical in informing professional societies and local experts for the need to develop structured recommendations (ie, tables, flowcharts) rather than narrative text when publishing guidelines and evidence. Another factor is the training of the CELS author. Previous studies have focused on comparing computer scientists to physicians when authoring guideline artifacts and conclude that they perform better when working together.^23^ In our particular domain, we demonstrate that diagnostic radiology guidelines are more accurately represented by specialty-trained physicians.

Several reasons have been proposed for variations in artifact representation. These include (1) differences in the representation of detail (eg, insufficient detail); (2) differences in the organization of medical concepts; and (3) differences in encoding temporal information (eg, insufficient temporal information).^23^ We noted these 3 factors in those CELS representations that were inaccurate compared with gold standard. For instance, detail representation varies in the following atom for a low back pain recommendation: “*adequate conservative treatment without improvement*” versus “*physical therapy/chiropractic evaluation in preceding 60 days with no improvement* OR *follow-up evaluation and management in preceding 28–60 days with no improvement*.” The second representation is more detailed and has 2 atoms instead of one. Authors with more relevant experience may provide more detailed as well as more granular representations. Other examples of inaccurate artifact representations are shown in Table 4.

**Table 4.** Examples of inaccurate CELS representation

CELS evidence structure	Clinical domain	Reason for inaccurate representation	Author representation	Gold standard representation
Structured	Low back pain	Insufficient detail	IF “adequate conservative treatment without improvement”	IF “physical therapy/chiropractic evaluation in preceding 60 days with no improvement” OR “follow-up evaluation and management in preceding 28–60 days with no improvement”
Semistructured	Headache	Difference in the organization of medical concepts	IF “headache” AND NOT “posttraumatic”	IF “atraumatic headache”
Semistructured	Headache	Insufficient temporal information	IF “headache” AND NOT “posttraumatic”	IF NOT “posttraumatic headache (ie, head trauma in the previous 4 weeks)”
Structured	Headache	Other reason (eg, Incomplete representation) AND difference in the organization of medical concepts	IF “existing headache disorder” AND “increase in headache frequency” OR “increased headache severity” OR “increased headache duration” THEN “MRI brain without contrast”	IF “existing headache disorder” AND “clinical progression” AND “Significant increase in headache frequency, severity or duration” THEN “MRI brain without contrast”

Nevertheless, a goal of knowledge representation is to capture the clinical intent of evidence recommendations.^30^ Thus, it was essential to verify whether CELS representation accuracy mirrors the semantic intentions of the author(s) of the evidence.^30^ Even though some atoms were less detail-specific and were therefore inaccurately represented, clinical intent may be similar when assessed by experts. We demonstrated, however, that semistructured evidence represented less accurately as CELS are also significantly less similar to the clinical intent of the textual evidence when assessed by clinical experts.

This study did have a limitation; although clinical experts assessed the similarity of CELS to textual evidence and the clinical intent of the evidence, we did not determine impact on use as CDS artifacts during an actual implementation.

## CONCLUSION

Authoring instructions with an educational intervention significantly improved CELS representation with a 3-fold increase in accuracy. CELS represented from structured evidence had higher representation accuracy compared with semistructured evidence. Similarly, specialty-trained authors had higher accuracy when representing structured evidence. However, CELS representation remains a challenging task with only 24% of CELS represented accurately.

## AUTHOR CONTRIBUTIONS

All coauthors contributed to the conception and design of this project. RL, ME, LC, IG, AL, AZ, and RK specifically contributed to data acquisition and analysis. All coauthors contributed significant intellectual content during the article preparation, approved the final version, and accept accountability for the overall integrity of the research process and the article.
